# The first Sri Lankan family with Dent disease-1 due to a pathogenic variant in the *CLCN5* gene: a case report

**DOI:** 10.1186/s13104-017-2881-5

**Published:** 2017-10-30

**Authors:** Randula Ranawaka, Nirmala Dushyanthi Sirisena, Kavinda Chandimal Dayasiri, Andrea G. Cogal, John C. Lieske, Manoji Prabashini Gamage, Vajira H. W. Dissanayake

**Affiliations:** 1 Department of Paediatrics, Faculty of Medicine, University of Colombo, Kynsey Road, Colombo 8, Sri Lanka; 2 Human Genetics Unit, Faculty of Medicine, University of Colombo, Colombo 8, Sri Lanka; 3 Professorial Paediatric Unit, Lady Ridgeway Hospital for Children, Colombo 08, Sri Lanka; 4 Rare Kidney Stone Consortium/Dent Disease Program, Mayo Clinic Division of Nephrology and Hypertension, Rochester, MN USA; 5 Nutrition Unit, Lady Ridgeway Hospital for Children, Colombo, Sri Lanka

**Keywords:** Dent disease-1, Genetics, Low molecular weight proteinuria, Renal tubular disorder, X-linked recessive

## Abstract

**Background:**

Dent disease-1 is a rare X-linked recessive renal tubular disorder caused by pathogenic variants in the chloride voltage-gated channel 5 (*CLCN5*) gene. It is characterized by low-molecular-weight proteinuria, hypercalciuria, nephrocalcinosis, nephrolithiasis and progressive renal failure. This is the first report of a *CLCN5* pathogenic variant in a Dent disease-1 family of Sri Lankan origin, and it highlights the value of genetic evaluation in children with refractory proteinuria.

**Case presentation:**

A 2-year-old boy with non-nephrotic range proteinuria was referred for evaluation. His maternally related 24-year-old uncle had been investigated for similar features at the age of 14 years and his renal histology had shown few sclerosed glomeruli. He remained asymptomatic apart from proteinuria. Biochemical investigation of the child showed β-2 microglobulinuria and hypercalciuria. After providing pre-test counseling and obtaining written informed consent, the child, his mother and maternal uncle underwent genetic testing for confirmation of the clinically suspected diagnosis of Dent disease-1. Both the child and his maternal uncle were found to be hemizygous for a nonsense pathogenic variant in exon 9 of the *CLCN5* gene [NM_000084.4; c.1399C>T; rs797044811] which results in a stop codon at residue 467, leading to a truncated non-functional protein [NP_000075.1; p.R467X]. His mother was confirmed to be an unaffected heterozygous carrier for the same variant. Following confirmation of the diagnosis our patient was started on thiazide diuretics and potassium citrate.

**Conclusions:**

Even though the typical phenotype of Dent disease-1 often enables a clinical diagnosis to be made, less severe sub-clinical cases may go undiagnosed. The underlying diagnosis may be missed especially in children who are treated for non-minimal change nephrotic syndrome with steroids. This case highlights the need for tubular proteinuria to be considered in the differential diagnosis of children with refractory proteinuria and for appropriate genetic evaluation to be done to confirm the precise underlying diagnosis in such cases.

## Background

Dent disease [MIM number 300009] is a rare X-linked recessive renal tubular disorder characterized by manifestations of proximal tubule dysfunction which is seen exclusively in males. The tubular dysfunction leads to low-molecular-weight (LMW) proteinuria, hypercalciuria, nephrocalcinosis, nephrolithiasis, rickets and progressive renal failure [1]. Dent and Friedman described the first reports of Dent disease in 1964 in two unrelated English boys with rickets associated with renal tubular damage characterized by hypercalciuria, hyperphosphaturia, proteinuria, and aminoaciduria [2]. Evidence on the global prevalence of Dent disease is not available and the limited data available suggest [3] that it has been documented in around 250 families to date.

Dent disease-1 is caused by pathogenic variants in the chloride voltage-gated channel 5 (*CLCN5*) gene located on chromosome Xp11.23, which encodes the kidney specific electrogenic chloride/proton (Cl^−^/H^+^) exchange transporter ClC-5. The encoded protein is primarily localized to endosomal membranes and may function to facilitate albumin uptake by the renal proximal tubules. Dent disease-2 [MIM number 300555] is caused by pathogenic variants in the inositol polyphosphate-5-phosphatase (*OCRL1*) gene and is also associated with Lowe syndrome. The clinical hallmark of Dent disease-1 is LMW proteinuria which is seen in all affected males and obligate female carriers. It is associated with generalized proximal renal tubular dysfunction and often complicated with hypophosphatemic rickets [4], nephrolithiasis, nephrocalcinosis and end-stage renal disease. The disease usually presents in childhood or early adult life, and most clinically affected cases are males, while female carriers are usually asymptomatic. Rarely, heterozygous females may manifest clinically significant kidney disease resulting from skewed X-chromosome inactivation. This is the first report of a *CLCN5* pathogenic variant in a Dent disease-1 family of Sri Lankan origin, and it highlights the value of genetic evaluation in children with refractory proteinuria.

## Case presentation

A 2-year-old boy was referred to a paediatric nephrologist for evaluation of non-nephrotic range proteinuria. He did not have hematuria, body swelling or features of vasculitis. His 24-year-old maternal uncle had been evaluated for the same presentation 10 years ago and a renal biopsy revealed non-specific changes with a few sclerosed glomeruli. However, he remained asymptomatic apart from the proteinuria.

Physical examination of the child did not show objective weight gain, ascites, pleural effusion or hypertension. Blood pressure was normal at 85/51 mmHg. He had normal serum albumin concentration despite having an elevated urine protein/creatinine ratio of 176 mg/mmol/l which is in the non-nephrotic range. Trace amounts of albumin with normal globulin fractions were identified by urine protein electrophoresis. Urine β-2 microglobulin concentration was elevated at > 500 ng/ml (normal range < 300 ng/ml). Serum protein electrophoresis revealed high-normal albumin concentration without abnormalities. The 24-h urinary calcium (1.58 mmol/day) and adjusted urinary calcium excretion (0.14 mmol/kg/day; normal range < 0.06 mmol/kg/day) were both elevated. He had a normal serum phosphate level and fractional excretion of urinary phosphate was normal. Urinary tract ultrasound was normal and showed no evidence of nephrocalcinosis. A diagnosis of Dent disease was clinically suspected based on the β-2 microglobulinuria, hypercalciuria and the family history, and thus screening was offered to the family. Table 1 shows the biochemical profile of the child.

**Table 1:** Results of the biochemical investigations performed in the child

Investigation	Result	Normal range
Serum albumin	44 g/l	34–54 g/l
Serum sodium	135 meq/l	135–145 meq/l
Serum potassium	3.6 meq/l	3.5–5.0 meq. l
Serum cholesterol	4.98 mmol/l	3.6–5.7 mmol/l
Blood urea	2.4 mmol/l	1.5–3 mmol/l
Urine albumin	+ 1/trace	
Urine red blood cells	0–1/hpf	< 10 cells/hpf
Serum creatinine	33 µmol/l	< 44 µmol/l
Adjusted urinary phosphate	0.59 mmol/kg/day	0.48–0.64 mmol/kg/day
Serum phosphate	1.48 mmol/l	1.45–1.78
Serum protein electrophoresis		
Albumin	46 g/l	35.00–45.00
Alpha 1	1.69	1.00–3.00
Alpha 2	6.00	6.00–10.00
Beta	5.4	7.00–11.00
Gamma	6.92	8.00–16.00

After providing pre-test counseling and obtaining written informed consent, the child, his mother and maternal uncle underwent genetic testing for Dent disease at the Rare Kidney Stone Consortium (Mayo Clinic, Rochester, MN, USA). Individual exons with flanking intron sequences of the *CLCN5* gene were amplified and sequenced from DNA extracted from peripheral venous blood. The primers and conditions for thermal cycling are available upon request. Both the child and his maternal uncle were found to be hemizygous for a nonsense pathogenic variant in exon 9 of the *CLCN5* gene [NM_000084.4; c.1399C>T; rs797044811], which results in a stop codon at residue 467, leading to a truncated non-functional protein [NP_000075.1; p.R467X]. This confirmed the diagnosis of Dent disease-1 in the proband and his uncle. His mother was also confirmed to be an unaffected heterozygous carrier for the same variant. This pathogenic variant has previously been reported in association with Dent disease-1 in the published literature [5].

Following confirmation of the diagnosis our patient was started on thiazide diuretics and potassium citrate. After 1 month of therapy his adjusted urinary calcium excretion was 0.08 mmol/kg/day (normal range < 0.06 mmol/kg/day).

## Discussion and conclusions

Dent disease is an X-linked form of progressive renal disease characterized by hypercalciuria and proximal tubular dysfunction. The majority of patients remain asymptomatic throughout childhood. This is the first report of a *CLCN5* pathogenic variant in a Dent disease-1 family of Sri Lankan origin. Recent reports from India described a severe clinical phenotype among patients having *CLCN5* Dent disease [6]. The adult patient described in this report remained asymptomatic with normal renal functions at the time of his diagnosis at 24 years of age. Focal glomerular sclerosis is a common histological finding in Dent disease [7, 8] as seen in the proband’s uncle.

High clinical vigilance is important in investigating children with proteinuria since tubulopathy may be misinterpreted for glomerular proteinuria. LMW proteinuria in Dent disease can be confirmed by measuring increased urinary excretion of one of the following: α1- and β2-microglobulins, retinol-binding protein (RBP), Clara cell protein, and vitamin D binding protein (reported in 99% of affected males) [9]. Hypercalciuria and nephrocalcinosis are reported in 95 and 75% of affected males, respectively.

To date about 250 affected families with Dent disease-1 have been reported, and about 174 different *CLCN5* pathogenic variants have so far been identified, the majority being either nonsense or missense variants (69%) [3]. The phenotype of our patients was similar to that previously reported in patients with the same pathogenic variant in the *CLCN5* gene [5]. Genetic heterogeneity exists in the *CLCN5* variants and clear-cut genotype–phenotype correlations have yet to be established [10, 11]. This emphasizes the value of establishing the genetic diagnosis in all clinically suspected cases.

Management of Dent disease is supportive and includes control of hypercalciuria with thiazide diuretics [12] and treatment to prevent rickets as needed. Potassium citrate may also play a role in ameliorating kidney stone risk and possibly nephrocalcinosis and chronic kidney disease (CKD) progression [13]. Progression to CKD and renal failure often occur between the third and fifth decades of life in 30–80% of male patients [14].

Both proximal renal tubular acidosis [15] and focal glomerulosclerosis [16] are associated with Dent disease-1. Even though a typical phenotype characterized by LMW proteinuria, hypercalciuria, nephrocalcinosis, nephrolithiasis, rickets and progressive renal failure in various combinations often enables a clinical diagnosis, less severe sub-clinical cases may go under-diagnosed. Furthermore, it is likely that the underlying diagnosis of Dent disease may be missed especially in children who are treated for non-minimal change nephrotic syndrome with steroids. Such measures delay the correct diagnosis being made, but more importantly unnecessarily expose the child to the effects of immunosuppressant medication. It therefore bears emphasis that the possibility of tubular proteinuria needs to be considered in the differential diagnosis of children with refractory proteinuria, and appropriate genetic evaluation should be done to confirm the precise underlying diagnosis in such cases.

## Abbreviations

*CLCN5*: chloride voltage-gated channel 5 gene
Cl^−^/H^+^: chloride/proton
LMW: low-molecular-weight
*OCRL1*: inositol polyphosphate-5-phosphatase gene
